# Health Insurance Utilization and Its Impact: Observations from the Middle-Aged and Elderly in China

**DOI:** 10.1371/journal.pone.0080978

**Published:** 2013-12-06

**Authors:** Yu Wang, Yan Jiang, Yang Li, Xiaojun Wang, Chi Ma, Shuangge Ma

**Affiliations:** 1 School of Statistics and The Center for Applied Statistics, Renmin University of China, Beijing, China; 2 School of Public Health, Yale University, New Haven Connecticut, United States of America; 3 Humanities and Social Science College, Beijing Institute of Petrochemical Technology, Beijing, China; UNAIDS, Trinidad And Tobago

## Abstract

**Objective:**

In China, despite a high coverage rate, health insurance is not used for all illness episodes. Our goal is to identify subjects’ characteristics associated with insurance utilization and the association between utilization and medical expenditure.

**Methods:**

A survey was conducted in January and February of 2012. 2093 middle-aged and elderly subjects (45 years old and above) were surveyed.

**Results:**

Heath insurance was not utilized for 12.6% (inpatient), 53.3% (outpatient), and 72.6% (self-treatment) of disease episodes. Subjects’ characteristics were associated with insurance utilization. Inpatient and outpatient treatments were expensive. In the multivariate analysis of outpatient treatment expenditure, insurance utilization was significantly associated with higher treatment cost, lost income, and gross total cost.

**Conclusion:**

Utilization of health insurance may need to be improved. Insurance utilization can reduce out-of-pocket medical expenditure. However, the amount paid by the insured is still high. Policy intervention is needed to further improve the effectiveness of health insurance.

## Introduction

In the recent years, China has experienced significant development in healthcare. In 2009, the Chinese government committed $125 billion to healthcare reform. This reform has affected multiple facets of health care delivery, including health insurance, primary care, hospital management, medication, and public health [1]. This article is focused on health insurance utilization and its association with medical expenditure.

China is approaching fast the goal of providing universal health insurance coverage by 2020. The current insurance system is composed of basic and commercial insurance. The basic health insurance consists of three schemes for different populations and takes different forms in rural and urban areas. In principle, every citizen is covered by one basic insurance scheme. In the rural area, the new rural cooperative medical care system (NCMS) is a mutual-help and risk-pooling health protection system [2]. By 2010, the number of NCMS participants had reached 835 million, accounting for 96.3% of the total rural population [3]. In the urban area, there are two schemes. The urban employee basic medical insurance for urban employed (UEBMI) requires the enrollment of all urban employees. The urban resident basic medical insurance for urban residents (URBMI) covers urban residents who are not employed as well as children [4]. It is estimated that over 90% of China’s population are now covered by basic health insurance. In comparison, commercial health insurance is less developed. It mainly targets the upper class, complementing basic health insurance [4]. It now serves about 7% of China’s population and has grown about 27% annually over the past decade.

A large number of studies have been conducted on China’s health insurance. Barber and Yao [5] conducted a comprehensive review and discussed problems including reliance on local government capacity, reimbursement ceilings and rates, incentive for unnecessary care, and waste in the design of programs. A survey study conducted in 2011 in three large cities and their surrounding rural areas showed that smaller households, higher income, lower expense, presence of at least one inpatient treatment, and living in rural areas were significantly associated with a lower coverage rate [6]. Chen and Yan [7] investigated the demand for URBMI and identified the associated factors including income, health condition, age, and health risk behaviors. Wang and others [8] conducted a survey in six villages and identified income and health condition as important factors for pursuing health insurance (participating in basic health insurance or purchasing commercial insurance) and utilizing services. The relationship between health insurance coverage and medical expenditure and their impacts on households’ overall wellbeing have also attracted extensive attention. It has been shown that although the overall health insurance coverage depth – scope and percentage of expense reimbursement – has been increasing over the years, it is still insufficient and varies significantly [1]. In addition, copayments and deductibles remain high. As a result, the Chinese are paying 40% or more of all health costs as either premiums or OOP (out of pocket) payments. A recent study found that households participated in the NCMS were less likely to become impoverished [9]. Fang and others [6] suggested that even for households with insurance coverage, there was considerable OOP cost, particularly for those with inpatient treatments and/or chronic diseases. Li and others [10] found that families enrolled in URBMI or UEBMI had lower rates of catastrophic health expenditure than those enrolled in NCMS. A few other aspects of health insurance have also been studied. For example, Lei and Lin [11] investigated the service and health outcomes of NCMS. Qiu and others [12] studied the rural to urban migration and its impact on NCMS. Wang and others [13] identified an adverse selection problem in an early voluntary form of NCMS.

This study has been motivated by the following considerations. First, in the literature, there are many studies on insurance coverage. However, research on utilization, that is whether insurance is actually used for a specific disease episode, remains limited. Utilization provides a more effective and more direct measure of effectiveness than coverage. In a study recently conducted in Vietnam, Nguyen and others [14] showed that people that had but did not use insurance behaved significantly differently from those that used insurance. With a close-to-perfect insurance coverage rate in China, the study of utilization can be informative to insurance agencies and the insured. Second, the existing studies can also be limited in that they often focus on a specific type of insurance, for example NCMS only. To provide a more comprehensive description, multiple types of health insurance need to considered together. Third, when investigating the association between health insurance and medical expenditure, many studies have focused on the total cost. Investigating each category of cost (treatment, transportation, medicine, etc; more details are provided below in the Data Collection section) may provide insights beyond total cost. Fourth, many studies have used various census data published by the central and local governments [10], [15]. Such data may have two limitations. One is that there is usually insufficient information on personal behaviors and outcomes. In addition, Yip and others [16] argued that “… independent, outcome-based monitoring and evaluation by a third-party are essential … to make officials and providers accountable.”. Last, with the fast development and system-wide reform started in 2009, observations made in early studies may not hold [17]. There is a need for an updated description.

This study has two main objectives. The first is to identify subjects’ characteristics associated with insurance utilization. The second is to investigate whether insurance utilization is associated with the amount of each category of medical expenditure. For both objectives, analysis is conducted for inpatient, outpatient, and self-treatment separately. To this end, we conducted a survey, and collected and analyzed detailed micro data. This study is among the few that focuses on insurance utilization in China and may provide valuable insights beyond the existing studies.

## Methods

### Data Collection

An in-house survey was conducted in January and February of 2012. The study was approved by a research ethics review committee at Renmin University of China (RUC).

Joint with other RUC researchers, we have been conducting large-scale, longitudinal survey studies on the wellbeing and medical and social security of the middle-aged and elderly (45 years old and above). This age group can be more vulnerable to illness conditions and in more need of health insurance protection [18]. Samples of the existing studies have been randomly selected, covering the majority of municipalities and provinces in mainland China. Samples of this study were randomly selected from the existing samples, due to convenience and cost considerations. In sample selection, stratification by area, region, and GDP level was considered in an attempt to achieve representativeness. The resulted samples were from 152 cities of 25 provinces and municipalities. The following provinces and municipality were not included because of resource limitations: Shanghai, Guangxi, Hainan, Xizang, Qinghai, and Ningxia.

At the beginning of each survey, the interviewer introduced the purpose of survey, the nature of questions to be asked, and how the collected information would be used. Each interviewee was asked to sign an informed consent form. Basic information was collected to determine inclusion. An interviewee would be excluded if he/she had not participated in the existing medical and social security studies, or was younger than 45, or could not provide reliable information on illness conditions and expenditure. Less than ten interviewed subjects were excluded because of not being in the existing studies or younger than 45. The survey response rate was 86%. The main reasons for refusal included “not comfortable with providing certain information”, “too busy to complete the survey”, and “concerns over how the survey results will be presented”.

Some questions reflected the status at the time of survey (such as age, gender, marital status, education, and occupation), whereas others described the “accumulation” over a period of twelve months prior to survey (such as illness conditions, income, and medical expense). Two categories of data were collected. The first included demographic and personal characteristics, such as gender, age, marital status, education, occupation, household income, personal income, general physical condition, area, region, and access to health facility. Urban or rural area was defined by “Hukou”, a household registration issued by the central government. The whole surveyed region was classified into eastern, western, and central. The eastern region is in general more developed and has a higher economic status than the western and central regions. The second category of data described illness conditions. Detailed information, including grade of hospital used, whether insurance was used, and medical expense, was collected on inpatient treatment, outpatient treatment, and self-treatment separately. A person was considered ill if he/she was diagnosed by a healthcare professional, experienced discomfort, or was unable to pursue usual activities. Inpatient treatment was defined as an appointment, procedure and/or treatment requiring an overnight stay in a health facility. Outpatient treatment was defined similarly but without an overnight stay. Self-treatment was defined as the scenario where an individual used unprescribed drugs or other medical approaches to treat untreated and often undiagnosed medical conditions. It remains an important and common health-seeking behavior in China [19], [20]. Hospital-based healthcare services are of better quality, whereas self-treatment is more affordable in terms of money and time.

Information on whether a subject was covered by at least one type of health insurance was collected. In addition, we collected information on whether insurance was actually used for illness episodes. For inpatient and outpatient treatments, insurance can cover partial cost of examination, treatment, medicine, and others. For self-treatment, insurance can pay over-the-counter medicine. For medical expense, data collected included the cost of treatment, transportation, food and accommodation, medicine/supplies, unofficial gift (to employees of health facilities, escorts, and caretakers), and lost income. This study differs from some of the existing ones by accounting for unofficial gift and lost income, which are direct consequences of illness conditions.

### Data Analysis

Initial analyses were performed on all persons surveyed. As the main interest in this paper is on the impact of health insurance utilization, subsequent analyses were only performed on persons who had health insurance coverage and at least one disease episode. This analysis strategy is in line with Nguyen et al. [14] and others.

Due to consideration on resource, we had sampled multiple members from the same families, if they were all included in the existing survey studies. Frailty models were used to accommodate within-family correlations. In the univariate analysis of subjects’ characteristics and medical expenditure, we compared disease episodes that used insurance against those that did not. P-values were computed from Chi-squared tests for categorical variables and t tests for continuous variables. Multivariate analysis was then conducted, accounting for confounding effects. As there are only a moderate number of variables and a large sample size, all variables, no matter significant or not in univariate analysis, were included in multivariate analysis. In the first set of analysis, we searched for factors associated with insurance utilization. Here the response variable was the binary insurance utilization status. Logistic regression analysis was conducted, and the adjusted odds ratios (aOR) and p-values were computed. In the second set of analysis, we regressed medical expense (each category, gross total, and OOP) on insurance utilization, adjusting for confounding effects. Linear regression was conducted. The estimated regression coefficients and their significance level were computed. Analysis was conducted using S-Plus Version 8.2 (TIBCO Software Inc.).

The full dataset is now password-protected and securely stored at RUC. The questionnaire and a subset of deidentified data are available at http://stat.ruc.edu.cn/a/kexueyanjiu/keyanchangyongwenjian/2013/0827/618.html. Per funding regulations, the complete deidentified data will be publicly available on July 1^st^, 2014. Prior to that date, access to the data needs to be applied and approved on a case-by-case basis.

## Results

### Characteristics of the Whole Cohort

2,093 subjects from 711 urban and 441 rural households were surveyed. There were 1,059, 515, and 518 subjects from the eastern, central, and western regions, respectively. Among them, 1,053 were male. The mean age was 57.0. 1,928 subjects were covered by at least one type of insurance and had at least one disease episode. During a period of twelve months, there were 373 (inpatient), 1,308 (outpatient), and 1,568 (self) treatments, among which 47 (inpatient, 12.6%), 697 (outpatient, 53.3%), and 1,139 (self, 72.6%) did not use insurance.

### Characteristics of Disease Episodes

Subjects’ characteristics for disease episodes that used and did not use insurance were summarized in Table 1. For patients that did not use insurance for inpatient treatments, 61.7% were female; however, the gender difference was not significant (p-value 0.086). The gender differences were even smaller for outpatient and self-treatments. For inpatient treatment, patients that used insurance were older (p-value 0.026). The association was reversed for self-treatment, although it was only borderline-significant (p-value 0.053). No association was observed for marital status. The associations between education level and insurance utilization for outpatient and self-treatments were significant. For occupation, the p-values were 0.069, <0.001, and <0.001 for the three types of treatments, respectively. For outpatient treatment, income level had significant associations, with a higher income corresponding to a higher probability of using insurance. Physical condition did not have a significant association. Compared to eastern, subjects in the central and western regions were less likely to use insurance for outpatient treatment. For outpatient and self-treatments, urban residents were more likely to use insurance. For outpatient treatment, the type of hospital used for treatment also had a significant association (p-value<0.001). In particular, those who used grade III hospitals were more likely to use insurance. The survey also collected information (distance, type, and travel time) on the nearest hospital, which may partly measure the access to healthcare [14]. It is noted that the nearest hospital was not necessarily the one used for treatment. For outpatient and self-treatments, the associations for the type of the nearest hospital and travel time were significant.

**Table 1:** Summary statistics on disease treatments with different insurance utilization status.

	Inpatient		Outpatient		Self-treatment	
Insurance utilization	Yes	No	Yes	No	Yes	No
Sample size	326	47	611	697	429	1139
**Gender**						
Female	157 (48.3)	29 (61.7)	314 (51.5)	361(51.8)	223 (52.0)	574 (50.4)
Male	168 (51.7)	18 (38.3)	296 (48.5)	336(48.2)	206 (48.0)	564 (49.6)
	P = 0.086		P = 0.909		P = 0.586	
**Age**	63.14±12.12	58.98±10.76	57.9±10.5	57.8±10.7	55.9±9.9	57.0±10.4
	P = 0.026		P = 0.872		P = 0.053	
**Marital status**						
Single/Divorced/Widowed	49 (15.1)	4 (8.5)	68 (11.1)	70 (10.1)	43 (10.0)	123 (10.8)
Married	276 (84.9)	43 (91.5)	542 (88.9)	626(89.9)	385 (90.0)	1016(89.2)
	P = 0.229		P = 0.523		P = 0.666	
**Education**						
No schooling	36 (11.1)	10 (21.3)	34 (5.6)	82 (11.8)	18 (4.2)	113 (10.0)
Primary	84 (26.0)	9 (19.1)	94 (15.5)	136(19.6)	53 (12.5)	213 (18.8)
Junior high	76 (23.5)	9 (19.1)	152 (25.1)	179(25.8)	85 (20.0)	295 (26.0)
Senior high	63 (19.5)	9 (19.1)	109 (18.0)	146(21.1)	91 (21.4)	216 (19.0)
Junior college and more	64 (19.8)	10 (21.3)	217 (35.8)	150(21.6)	178 (41.9)	297 (26.2)
	P = 0.337		P<0.001		P<0.001	
**Occupation**						
Governments	39 (12.1)	4 (8.5)	133 (22.1)	85 (12.4)	126 (29.8)	153 (13.6)
Enterprises	36 (11.2)	4 (8.5)	98 (16.3)	105(15.3)	99 (23.4)	192 (17.1)
Farmers	58 (18.0)	10 (21.3)	111 (18.4)	169(24.6)	45 (10.6)	265 (23.6)
Small private business	9 (2.8)	5 (10.6)	19 (3.2)	53 (7.7)	20 (4.7)	87 (7.7)
Others	25 (7.8)	4 (8.5)	33 (5.5)	61 (8.9)	20 (4.7)	108 (9.6)
Retired	112 (34.8)	10 (21.3)	163 (27.0)	116(16.9)	92 (21.7)	174 (15.5)
Unemployed	43 (13.4)	10 (21.3)	46 (7.6)	98 (14.3)	21 (5.0)	145 (12.9)
	P = 0.069		P<0.001		P<0.001	
**Per capita income (K RMB)**	32.5±67.4	23.0±16.0	39.9±72.3	25.2±28.9	35.2±54.4	30.0±47.4
	P = 0.352		P<0.001		P = 0.068	
**Household income(K RMB)**	91.1±200.4	73.5±53.9	114.8±207.0	78.2±86.3	99.7±156.4	89.3±120.6
	P = 0.559		P<0.001		P = 0.172	
**Physical condition**						
Healthy	46 (14.1)	8 (17.0)	208 (34.0)	223(32.0)	189 (44.1)	451 (39.6)
Just so-so	110 (33.7)	23 (48.9)	258 (42.2)	319(45.8)	170 (39.6)	480 (42.1)
Slightly sick	84 (25.8)	11 (23.4)	83 (13.6)	101(14.5)	49 (11.4)	132 (11.6)
Sick	59 (18.1)	3 (6.4)	49 (8.0)	37 (5.3)	17 (4.0)	53 (4.7)
Seriously sick	27 (8.3)	2 (4.3)	13 (2.1)	17 (2.4)	4 (0.9)	23 (2.0)
	P = 0.124		P = 0.257		P = 0.346	
**Region**						
Eastern	161 (49.4)	22 (46.8)	396 (64.8)	313(44.9)	208 (48.5)	549 (48.2)
Central	69 (21.2)	13 (27.7)	99 (16.2)	176(25.3)	104 (24.2)	291 (25.5)
Western	96 (29.4)	12 (25.5)	116 (19.0)	208(29.8)	117 (27.3)	299 (26.3)
	P = 0.588		P<0.001		P = 0.844	
**Area**						
Rural	128 (39.4)	21 (44.7)	186 (30.4)	342(49.1)	76 (17.7)	527 (46.3)
Urban	197 (60.6)	26 (55.3)	425 (69.6)	354(50.9)	353 (82.3)	611 (53.7)
	P = 0.489		P<0.001		P<0.001	
**Type of hospital used for treatment**						
Grade I	47 (14.6)	4 (8.7)	192 (33.4)	228(34.7)	–	–
Grade II	105 (32.5)	18 (39.1)	134 (23.3)	173(26.3)	–	–
Grade III	165 (51.1)	22 (47.8)	231 (40.2)	192(29.2)	–	–
Private	6 (1.9)	2 (4.3)	18 (3.1)	65 (9.9)	–	–
	P = 0.429		P<0.001			
**Distance to the nearest hospital (meters)**						
< = 1000	256 (78.8)	34 (72.3)	474 (77.6)	509(73.3)	324 (75.5)	823 (72.4)
1001–5000	64 (19.7)	10 (21.3)	125 (20.5)	176(25.4)	99 (23.1)	297 (26.1)
> = 5001	5 (1.5)	3 (6.4)	12 (2.0)	9 (1.3)	6 (1.4)	17 (1.5)
	P = 0.093		P = 0.080		P = 0.454	
**Type of the nearest hospital**						
Grade I	131 (40.3)	15 (31.9)	272 (44.7)	322(46.2)	149 (34.7)	515 (45.3)
Grade II	68 (20.9)	8 (17.0)	118 (19.4)	109(15.6)	90 (21.0)	217 (19.1)
Grade III	65 (20.0)	16 (34.0)	136 (22.3)	134(19.2)	126 (29.4)	208 (18.3)
Private	38 (11.7)	7 (14.9)	41 (6.7)	83 (11.9)	40 (9.3)	108 (9.5)
Others	23 (7.1)	1 (2.1)	42 (6.9)	49 (7.0)	24 (5.6)	89 (7.8)
	P = 0.154		P = 0.009		P<0.001	
**Travel time to the nearest hospital (minutes)**						
< = 5	139 (42.9)	20 (42.6)	293 (48.2)	295(42.4)	212 (49.4)	496 (43.7)
6–10	111 (34.3)	16 (34.0)	199 (32.7)	216(31.1)	139 (32.4)	356 (31.3)
11–30	69 (21.3)	10 (21.3)	111 (18.3)	176(25.3)	78 (18.2)	269 (23.7)
> = 31	5 (1.5)	1 (2.1)	5 (0.8)	8 (1.2)	0	15 (1.3)
	P = 0.993		P = 0.016		P = 0.006	

For a categorical variable, count (percentage); For a continuous variable, mean ± standard deviation.

### Characteristics of Medical Expenditure

Table 2 summarizes medical expense for episodes that used and did not use insurance. For inpatient and outpatient treatments, those that used insurance have a higher treatment cost. The mean differences are 7033.9RMB (p-value 0.036) and 1203.3RMB (p-value<0.001), respectively (1RMB = 0.16US$). In addition, for inpatient and outpatient treatments, the gross total cost is also significantly associated with insurance utilization. A closer examination of Table 2 suggests that such differences are mainly driven by the differences in treatment cost. After adjusting for insurance payment, the OOP costs for the two insurance utilization groups are not significantly different.

**Table 2:** Medical expenditure (in RMB) for disease treatments with different insurance utilization status.

	Inpatient		Outpatient		Self-treatment	
Insurance utilization	Yes	No	Yes	No	Yes	No
**Treatment**	19237.1±41266.8	12203.2±16569.1	2461.8±4950.7	1258.5±3285.6	815.0±1646.1	666.1±1443.6
	P = 0.036		P<0.001		P = 0.086	
**Transportation, food,** **accommodation**	2632.9±10911.4	2400.0±3869.8	244.8±2743.1	77.2±266.1	–	–
	P = 0.885		P = 0.139			
**Medicine/supplies**	1652.7±11468.4	1393.6±3829.4	428.2±1219.4	425.8±1634.9	–	–
	P = 0.887		P = 0.976			
**Unofficial gifts**	438.0±2259.8	497.8±1675.5	41.1±312.2	34.7±470.9	–	–
	P = 0.863		P = 0.783			
**Lost income**	2183.9±17248.7	2184.1±4590.1	195.4±775.5	204.6±924.7	121.0±566.0	147.3±964.0
	P>0.999		P = 0.851		P = 0.611	
**Gross total cost**	23364.5±43412.8	15667.4±14381.0	3509.9±7379.4	2022.5±5335.8	907.7±1758.8	779.6±1590.9
	P = 0.022		P<0.001		P = 0.194	
**Paid by insurance**	13863.0±34507.8	0	1811.8±4010.3	0	492.5±758.2	0
**Out-of-pocket cost**	12499.1±30653.4	15667.4±14381.0	1880.4±5561.0	2025.3±5339.4	785.8±1878.9	796.7±1604.0
	P = 0.511		P = 0.647		P = 0.933	

In each cell, mean ± standard deviation.

Another observation is that, even with insurance, illness is still expensive. This observation is in line with the literature [21]. On average, the OOP cost for inpatient treatment is 12,499RMB (insurance used) and 15,667RMB (insurance not used). The OOP cost for outpatient treatment is 1,880RMB (insurance used) and 2,025RMB (insurance not used). For the surveyed urban and rural subjects respectively, the mean annual income is 41.4K and 16.8K RMB. The inpatient and outpatient treatment costs make up a considerable percentage of the total income [22].

### Multivariate Regression Analysis of Insurance Utilization

Results are presented in Table 3. For inpatient treatment, education was significantly associated with insurance utilization. Specifically, with no school as the baseline, primary and junior high were significantly more likely to use insurance (odds ratios 4.635 and 6.586, respectively). The other two categories also have odds ratios greater than 1, although not significant at the 0.05 level. With government as the baseline for occupation, small private business had an odds ratio of 0.179 (p-value 0.055). With healthy as the baseline for physical condition, sick had an odds ratio of 4.102 (p-value 0.071).

**Table 3:** Multivariate logistic regression analysis of the prevalence of insurance utilization for inpatient, outpatient, and self-treatments.

	Inpatient		Outpatient		Self-treatment	
	aOR	*P*	aOR	*P*	aOR	*P*
**Gender (baseline: female)**						
Male	1.099	0.813	0.851	0.250	0.821	0.134
**Age (baseline: 45–50)**						
51–60	1.374	0.527	1.228	0.214	0.835	0.242
61–70	2.029	0.274	1.089	0.707	1.046	0.849
>70	3.157	0.106	0.936	0.805	0.760	0.308
**Marital status (baseline: single, divorced, widowed)**						
Married	0.355	0.140	0.749	0.218	0.927	0.738
**Education (baseline: no school)**						
Primary	4.635	0.016	1.722	0.068	1.292	0.428
Junior high	6.586	0.007	2.273	0.007	1.052	0.876
Senior high	3.986	0.064	1.698	0.108	1.214	0.568
Junior college and more	4.323	0.095	2.146	0.032	1.047	0.897
**Occupation(baseline: government)**						
Enterprises	1.205	0.817	0.591	0.023	0.637	0.016
Farmers	0.508	0.486	0.794	0.453	0.430	0.005
Small private business	0.179	0.055	0.272	0.000	0.368	0.001
Others	1.271	0.822	0.564	0.090	0.339	0.001
Retired	1.060	0.943	0.893	0.664	0.622	0.042
Unemployed	0.290	0.179	0.405	0.005	0.245	0.000
**Areas (baseline: rural)**						
Urban areas	0.652	0.470	1.635	0.021	3.048	0.000
**Regions (baseline: eastern)**						
Central	1.095	0.835	0.407	0.000	0.973	0.861
Western	1.843	0.201	0.467	0.000	1.216	0.201
**Household income (K RMB)**	1.002	0.589	1.001	0.359	1.000	0.513
**Personal income (K RMB)**	1.003	0.639	1.003	0.092	0.997	0.065
**Physical condition (baseline: healthy)**						
Just so-so	0.964	0.944	0.786	0.112	0.935	0.628
Slightly sick	1.696	0.384	1.065	0.769	1.079	0.728
Sick	4.102	0.071	1.901	0.025	0.892	0.725
Seriously sick	2.502	0.331	0.809	0.663	0.532	0.288
**Hospital used (baseline: grade I)**						
Grade II hospital	0.462	0.224	0.788	0.195	–	–
Grade III hospital	0.596	0.410	0.972	0.872	–	–
Private hospital	0.149	0.110	0.307	0.000	–	–

aOR: adjusted odds ratio; P: p-value.

For outpatient treatment, more factors are significantly associated with insurance utilization. With no school as the baseline, junior high and junior college and more were significant more likely to use insurance (odds ratios 2.273 and 2.146, respectively). The other two categories also had odds ratios greater than 1 (1.722 and 1.698, respectively), however, not significant. With government as the baseline, enterprise, small private business, and unemployed had lower probabilities of using insurance (odds ratios 0.591, 0.272, and 0.405, respectively). Urban subjects were more likely to use insurance (odds ratio 1.635). Subjects living in the central and western regions were less likely to use insurance (odds ratios 0.407 and 0.467, respectively). Personal income (in K RMB) had an odds ratio of 1.003 (p-value 0.092). Sick subjects were more likely to use insurance. In addition, subjects treated in private hospitals were less likely to use insurance (odds ratio 0.307).

For self-treatment, two factors were found significant. With government as the baseline, subjects with the other occupations were less likely to use insurance. The unemployed were the least likely to use insurance (odds ratio 0.245, p-value <0.001). In addition, urban subjects were more likely to use insurance (odds ratio 3.048).

### Multivariate Regression Analysis of Medical Expenditure

Detailed results are provided in Tables S1–S3. Multiple demographic and personal characteristics are found as significantly associated with the amount of medical expenditure. In Table 4, we provide the estimated coefficients and their p-values for insurance utilization. For inpatient treatment, the effect of insurance utilization is not significant for any individual expense category or overall. For outpatient treatment, after adjusting for the confounding effects, those that used insurance had significantly higher treatment cost (estimated difference 756.9RMB, p-value 0.005), higher lost income (estimated difference 92.5RMB, p-value 0.044), and higher gross total (estimated difference 1241.1RMB, p-value 0.002). For self-treatment, after adjusting for confounders, the effect of insurance utilization is not significant.

**Table 4:** Multivariate linear regression of medical expenditure (in RMB).

	Inpatient		Outpatient		Self-treatment	
	B	P	B	P	B	P
**Treatment**	2965.9	0.637	756.9	0.005	49.4	0.597
**Transportation, food, accommodation**	−211.4	0.904	167.9	0.197		
**Medicine/supplies**	−467.6	0.803	−67.4	0.440		
**Unofficial gift**	116.0	0.770	−9.9	0.727		
**Lost income**	−28.5	0.992	92.5	0.044	28.5	0.410
**Gross Total cost**	4280.5	0.550	1241.1	0.002	51.1	0.632
**Out of pocket cost**	−4590.8	0.387	−135.0	0.692	−141.9	0.287

B: regression coefficient for “insurance utilization = Yes”; P: p-value.

## Discussion

### Main Findings and Possible Interpretations

Among the surveyed subjects, 1,928 (92.1%) had at least one illness episode and were covered by at least one type of insurance. As the three types of treatments were considered for a period of twelve months, and as the surveyed subjects were middle-aged and elderly, it is not surprising that such a high proportion had illness episodes. It has been reported that nation-wide, over 90% of the population are covered by basic health insurance. As both basic and commercial insurance are considered in this study, the observed high coverage rate is also reasonable.

In the survey, 12.6% of the inpatient treatments and the majority of outpatient and self-treatments did not use insurance. A large number of factors may contribute to the status of insurance utilization. Our preliminary literature review did not suggest any regulation or circumstance that might prevent the utilization of health insurance. Conceptually, there is no benefit not utilizing insurance. Thus, the survey had been designed to identify subjects’ characteristics associated with utilization. Compared with outpatient and self-treatments, inpatient treatments are more expensive but less frequent. Thus patients can be more motivated to use insurance for inpatient treatments. Under the current healthcare system in China, utilizing insurance is not “automatic”. The reimbursement process can be lengthy and complicated, which may prevent patients from using insurance for some treatments. Multivariate regression analysis suggests that certain groups are less likely to use insurance, including for example subjects with no education, living in rural area, living in the central and western regions, and with lower income. In terms of socioeconomic status, these groups are less-advantaged. The association between education and insurance utilization has been noted in the literature [14], which suggested that failing to understand the insurance system and the complexity of reimbursement process could prevent the less-educated from using insurance. Occupation can be correlated with several other factors, particularly including socioeconomic status, education level, and income level. In addition, some occupations, in particular government, have supplementary health insurance, leading to an overall superior package. Small private businesses are mainly located in urban areas and in the eastern region. Compared with the rural areas and the western and central regions, the urban areas and eastern region are in general more developed, with people having higher income and education levels. The observed differences in utilization that are associated with socioeconomic status are in line with those made in a study conducted in the Gansu and Zhejiang provinces [18], which focused on the inequality caused by income and advocated that “China will need to reduce the unequal distribution of income and expand the coverage of its health insurance schemes”. The basic health insurance policies are relatively uniform across regions. However, the actual implementation may still differ. In addition, URBMI and NCMS are funded by the central government as well as local governments and the insured. The amount that local governments contribute to URBMI and NCMS and the quality of service may depend on the local economic conditions, which may also explain the observed regional differences. For physical condition, the “sick” category corresponds to serious diseases but not too serious to go through the utilization/reimbursement process. A higher cost would be expected from a more serious disease, providing a stronger motivation to use insurance.

The ultimate goal of health insurance is to improve the wellbeing of all insured. The identified group with less insurance utilization is potentially more vulnerable to the consequences of illness. The development of policy interventions, such as education and outreach programs, is needed to boost insurance utilization among that group. Compared to inpatient treatment, insurance is much less used for outpatient and self-treatments. Each episode of outpatient and self-treatment is less expensive (which partly explains why insurance is less used). However, they are more frequent, leading to a considerable total cost. In the literature, much attention has been paid to inpatient treatment. Our survey suggests that insurance utilization for outpatient and self-treatments deserves equal attention.

The observed medical expenditure is considerably higher than that in You and Kobayashi [15], which analyzed the 2004 China Health and Nutrition Survey and reported an average OOP expenditure of 502RMB. Such a difference can be explained by the fact that this study focused on the middle-aged and elderly who have higher medical expenses and by the fast economic growth that has caused medical expenditure to grow significantly. Table 2 shows high OOP costs, even for those that used insurance. Multiple factors contribute to this observation. First, some expenses such as transportation, food, accommodation, unofficial gifts, and lost income are not covered by insurance. Second, the insurance system is not sufficiently effective. Copayments and deductibles are still high. In addition, there are reimbursement ceilings. Our observation is in line which that in Liu [21], which reported that the average reimbursement rate of inpatient expense was as low as 48% for urban residents and 44% for rural residents. High OOP costs were also observed by Fang and others [6]. The high OOP cost poses a serious problem to the insured, in particular those with a low socioeconomic status. Data analysis suggests that insurance utilization is not statistically significantly associated with the cost of inpatient and self-treatment. Such a result differs from that in Nguyen et al. [14], which found that insurance can significantly lower medical cost. Many factors may have contributed to this difference, in particular the regional differences between China and Vietnam. In addition, examining data suggests that the observed medical cost have high variations. Thus although the actual cost for those used insurance and did not use insurance are quite different, the estimates are not statistically significant. There are a number of subjects with drastically high medical cost. Ideally, stratified analysis should be conducted, investigating the different behaviors for different illness conditions and medical cost groups. However, with a limited sample size, such an effort is not pursued. For self-treatment, the average cost is low, which may also contribute to the lack of significance. For outpatient treatment, insurance utilization is found to be significantly associated with higher treatment cost, higher lost income, and higher gross total cost. It should be noted that such observations do not mean insurance utilization causes higher costs. Patients with more serious diseases may expect higher costs, and hence are more likely to use insurance. Disease severity is likely to be the underlying driving factor for both higher cost and insurance utilization, causing them to be significantly associated.

### Limitations

The survey collected information for a period of twelve months. For a subject, illness conditions, particularly inpatient treatment, may vary from year to year. Although it was possible to collect information for a longer period, such an effort was not pursued because of the concern on recall error. The survey collected cross-sectional observational data. With such data, only association, not causality, can be inferred. Many published studies share the same limitation [6], [8], [14]. The nature of survey inevitably led to certain drawbacks, including limited information, possible recall bias, and others [23]. For example, the survey only collected information on whether insurance was used. There was no followup question on why insurance was not used. As discussed above and in the literature, possible reasons include misunderstanding of the insurance system, barriers in the reimbursement process, limited coverage depth, and others. However, without collecting additional information, we are not able to draw affirmative conclusions. This study collected data on inpatient, outpatient, and self-treatment separately, and can be more informative than those that measure illness and cost as a whole. However, a limitation is that there is no detailed information on disease. For example, it will be interesting to see if the associations observed in Table 3 still exist after adjusting for disease severity. This study may also be limited by having a moderate sample size, especially considering that the subjects were from 152 cities and that China has significant regional variations. All subjects were 45 years old or above. To provide a comprehensive description of the whole population, the younger cohort may be studied in the future.

## Conclusions

Research on China’s health insurance has attracted tremendous attention. In this study, we report empirical observations made in a survey recently conducted on insurance utilization and its impact. Demographic and personal characteristics are found as associated with insurance utilization. The findings may have important implications and can assist the development of intervention programs to further increase utilization and effectiveness. The analysis of medical expenditure leads to two main observations. The first is that insurance utilization is associated with some types of expenditure. More detailed investigations are needed to fully understand the mechanisms underlying such associations. In addition, it is found that even with health insurance utilization, the OOP cost is still high. High OOP cost may have a profound and long-term impact on a household’s wellbeing. More effective strategies are needed to improve coverage depth and reduce financial burden caused by illness.

## Supporting Information

Table S1 **Linear regression analysis of medical expenditure for inpatient treatment episodes.**
(DOCX)

Table S2 **Linear regression analysis of medical expenditure for outpatient treatment episodes.**
(DOCX)

Table S3 **Linear regression analysis of medical expenditure for self-treatment episodes.**
(DOCX)
