# Supplementary material for: Health Insurance Utilization and Its Impact: Observations from the Middle-Aged and Elderly in China
Source: PLoS One. 2013 Dec 6;8(12):e80978. doi: 10.1371/journal.pone.0080978 (PMC3855696; doi:10.1371/journal.pone.0080978)
Supplement: Table S1 — Linear regression analysis of medical expenditure for inpatient treatment episodes. (DOCX) [file pone.0080978.s001.docx]

**Table S1: Linear regression analysis of medical expenditure for inpatient treatment episodes.**

|  | **Treatment** | | **Transportation, food, accommodation** | | **Medicine/supplies** | | **Unofficial gift** | | **Lost income** | | **Gross Total cost** | | **Out of pocket cost** | |
| --- | --- | --- | --- | --- | --- | --- | --- | --- | --- | --- | --- | --- | --- | --- |
|  | **B** | ***P*** | **B** | ***P*** | **B** | ***P*** | **B** | ***P*** | **B** | ***P*** | **B** | ***P*** | **B** | ***P*** |
| **Gender (baseline: Female)** |  |  |  |  |  |  |  |  |  |  |  |  |  |  |
| Male | -4523.8 | 0.303 | -2499.0 | 0.041 | -1559.5 | 0.234 | 288.0 | 0.293 | 1263.5 | 0.538 | -339.7 | 0.945 | -113.4 | 0.976 |
| **Age group (baseline:45-50)** |  |  |  |  |  |  |  |  |  |  |  |  |  |  |
| 51-60 | -1204.1 | 0.847 | -1428.0 | 0.410 | -2340.7 | 0.210 | -522.4 | 0.180 | -3116.4 | 0.295 | -7001.2 | 0.323 | -7549.2 | 0.163 |
| 61-70 | 1778.5 | 0.811 | -1533.4 | 0.459 | -2294.2 | 0.302 | -82.8 | 0.859 | -4035.0 | 0.248 | -9479.5 | 0.258 | -7800.8 | 0.215 |
| >70 | 1406.2 | 0.857 | -2091.2 | 0.334 | -3041.3 | 0.191 | -785.9 | 0.107 | -3061.5 | 0.398 | -8939.4 | 0.308 | -11367.9 | 0.087 |
| **Marital status (baseline: Single/Divorced/Widowed)** |  |  |  |  |  |  |  |  |  |  |  |  |  |  |
| Married | 2183.3 | 0.742 | 561.5 | 0.761 | -175.2 | 0.930 | 195.1 | 0.638 | -819.2 | 0.792 | 4462.2 | 0.555 | 2036.6 | 0.722 |
| **Education (baseline: No school)** |  |  |  |  |  |  |  |  |  |  |  |  |  |  |
| Primary | -7715.4 | 0.308 | 2425.1 | 0.248 | 3764.3 | 0.095 | -1188.3 | 0.012 | -548.4 | 0.875 | -15845.4 | 0.062 | -3842.0 | 0.547 |
| Junior high | -5937.5 | 0.470 | -577.2 | 0.800 | 166.1 | 0.946 | -1590.6 | 0.002 | 3238.4 | 0.399 | -11139.1 | 0.229 | 2750.2 | 0.691 |
| Senior high | -12971.7 | 0.133 | 1306.5 | 0.585 | -1.1 | 1.000 | -1675.9 | 0.002 | -1196.2 | 0.768 | -15298.6 | 0.118 | -2744.2 | 0.711 |
| Junior college and more | -17744.8 | 0.070 | 588.5 | 0.828 | -481.5 | 0.869 | -1201.0 | 0.050 | -1250.2 | 0.783 | -21192.3 | 0.055 | -3705.5 | 0.654 |
| **Occupation (baseline: Governments)** |  |  |  |  |  |  |  |  |  |  |  |  |  |  |
| Enterprises | -3830.0 | 0.659 | -1870.6 | 0.439 | -461.8 | 0.859 | 205.0 | 0.705 | 608.9 | 0.882 | -2042.6 | 0.835 | 4696.9 | 0.530 |
| Farmers | -2510.7 | 0.813 | -3050.9 | 0.300 | -4465.0 | 0.158 | 668.0 | 0.314 | -2552.5 | 0.607 | 1426.4 | 0.905 | -840.8 | 0.926 |
| Small private business | -6761.2 | 0.601 | -1985.2 | 0.581 | -1399.8 | 0.717 | 653.7 | 0.418 | -1850.3 | 0.768 | -8164.4 | 0.585 | -1894.1 | 0.869 |
| Others | 4363.7 | 0.699 | 3892.9 | 0.212 | 5050.1 | 0.136 | 859.0 | 0.220 | 12235.5 | 0.021 | 20429.7 | 0.109 | 14563.2 | 0.133 |
| Retired | -897.9 | 0.914 | -2413.3 | 0.297 | -1525.0 | 0.539 | 339.9 | 0.513 | -147.4 | 0.970 | 1735.4 | 0.853 | 599.2 | 0.932 |
| No jobs | -12716.7 | 0.212 | -2497.3 | 0.377 | -4043.4 | 0.183 | 132.9 | 0.834 | -2797.6 | 0.558 | -6915.8 | 0.547 | -3289.3 | 0.704 |
| **Areas (baseline: rural)** |  |  |  |  |  |  |  |  |  |  |  |  |  |  |
| Urban areas | 5090.8 | 0.442 | -52.3 | 0.977 | -2529.2 | 0.200 | 446.9 | 0.279 | -4126.2 | 0.188 | 6741.2 | 0.371 | -3010.8 | 0.609 |
| **Regions (baseline: eastern)** |  |  |  |  |  |  |  |  |  |  |  |  |  |  |
| Central | -2148.3 | 0.684 | 2111.3 | 0.148 | 2689.1 | 0.087 | -275.3 | 0.401 | 3343.3 | 0.184 | 2556.2 | 0.672 | 6540.6 | 0.158 |
| Western | -12043.0 | 0.015 | -960.2 | 0.483 | -101.4 | 0.945 | -254.6 | 0.408 | -450.1 | 0.842 | -10444.1 | 0.055 | -3733.2 | 0.362 |
| **Physical condition (baseline: Healthy)** |  |  |  |  |  |  |  |  |  |  |  |  |  |  |
| Just so-so | -985.0 | 0.881 | 381.9 | 0.834 | 676.8 | 0.732 | -205.7 | 0.617 | 4089.7 | 0.200 | 3733.4 | 0.623 | 4577.2 | 0.430 |
| Slightly sick | 9042.7 | 0.198 | 1036.3 | 0.595 | 1075.9 | 0.610 | 291.0 | 0.509 | 1784.5 | 0.597 | 12096.7 | 0.134 | 7928.0 | 0.199 |
| Sick | 14027.6 | 0.063 | 4041.1 | 0.054 | 2837.8 | 0.210 | 92.3 | 0.845 | 3966.0 | 0.275 | 23972.1 | 0.006 | 17303.8 | 0.009 |
| Seriously sick | 40366.1 | <0.001 | 12592.0 | <0.001 | 10420.9 | <0.001 | 195.3 | 0.742 | 3491.0 | 0.438 | 29080.1 | 0.009 | 26207.5 | 0.002 |
| **Household income (1K Yuan)** | -20.618 | 0.098 | 3.514 | 0.309 | 3.628 | 0.328 | 0.778 | 0.317 | 3.100 | 0.587 | -12.5 | 0.360 | 0.166 | 0.987 |
| **Personal income (1K Yuan)** | 159.121 | <0.001 | -7.147 | 0.569 | -5.684 | 0.674 | -1.901 | 0.501 | -2.692 | 0.900 | 120.5 | 0.020 | 5.848 | 0.879 |
| **Hospital (baseline: Grade I)** |  |  |  |  |  |  |  |  |  |  |  |  |  |  |
| Grade Ⅱ hospital | 293.1 | 0.965 | 396.6 | 0.833 | -20.6 | 0.992 | 557.6 | 0.187 | -770.4 | 0.805 | -4331.2 | 0.558 | -2862.4 | 0.608 |
| Grade Ⅲ hospital | 18222.1 | 0.006 | 3047.5 | 0.094 | 2451.4 | 0.210 | 117.1 | 0.774 | 1863.4 | 0.541 | 16007.5 | 0.027 | 9255.7 | 0.088 |
| Private hospital | -6163.6 | 0.694 | 707.8 | 0.871 | 1899.5 | 0.685 | -136.6 | 0.889 | 2070.0 | 0.772 | -9584.2 | 0.575 | 63.9 | 0.996 |
| **Health insurance (baseline: not used)** |  |  |  |  |  |  |  |  |  |  |  |  |  |  |
| Yes | 2965.9 | 0.637 | -211.4 | 0.904 | -467.6 | 0.803 | 116.0 | 0.770 | -28.5 | 0.992 | 4280.5 | 0.550 | -4590.8 | 0.387 |
