# Supplementary material for: Health Insurance Utilization and Its Impact: Observations from the Middle-Aged and Elderly in China
Source: PLoS One. 2013 Dec 6;8(12):e80978. doi: 10.1371/journal.pone.0080978 (PMC3855696; doi:10.1371/journal.pone.0080978)
Supplement: Table S2 — Linear regression analysis of medical expenditure for outpatient treatment episodes. (DOCX) [file pone.0080978.s002.docx]

**Table S2: Linear regression analysis of medical expenditure for outpatient treatment episodes.**

|  | **Treatment** | | **Transportation, food, accommodation** | | **Medicine/supplies** | | **Unofficial gift** | | **Lost income** | | **Gross Total cost** | | **Out of pocket cost** | |
| --- | --- | --- | --- | --- | --- | --- | --- | --- | --- | --- | --- | --- | --- | --- |
|  | **B** | ***P*** | **B** | ***P*** | **B** | ***P*** | **B** | ***P*** | **B** | ***P*** | **B** | ***P*** | **B** | ***P*** |
| **Gender (baseline: Female)** |  |  |  |  |  |  |  |  |  |  |  |  |  |  |
| Male | 154.9 | 0.567 | 30.2 | 0.818 | 27.2 | 0.759 | 0.853 | 0.976 | 55.139 | 0.235 | 46.6 | 0.908 | 76.0 | 0.826 |
| **Age group (baseline: 45-50)** |  |  |  |  |  |  |  |  |  |  |  |  |  |  |
| 51-60 | 253.5 | 0.428 | 69.2 | 0.655 | -140.2 | 0.179 | -17.3 | 0.611 | -3.7 | 0.946 | 278.0 | 0.558 | -64.6 | 0.874 |
| 61-70 | -101.2 | 0.819 | -224.2 | 0.294 | 180.2 | 0.210 | -73.4 | 0.114 | -50.7 | 0.497 | 93.3 | 0.885 | -150.7 | 0.784 |
| >70 | -368.1 | 0.474 | -320.2 | 0.199 | -301.0 | 0.074 | -106.0 | 0.051 | -111.8 | 0.202 | -370.4 | 0.625 | -563.2 | 0.384 |
| **Marital status (baseline: Single/Divorced/Widowed)** |  |  |  |  |  |  |  |  |  |  |  |  |  |  |
| Married | -555.8 | 0.221 | 134.5 | 0. .544 | 7.3 | 0.961 | -137.5 | 0.005 | -261.1 | 0.001 | -252.2 | 0.714 | 139.7 | 0.810 |
| **Education (baseline: No school)** |  |  |  |  |  |  |  |  |  |  |  |  |  |  |
| Primary | -248.9 | 0.647 | 344.0 | 0.190 | -18.8 | 0.916 | -70.1 | 0.222 | -39.3 | 0.674 | 1131.5 | 0.161 | 1225.3 | 0.074 |
| Junior high | -943.1 | 0.093 | -88.3 | 0.745 | -214.1 | 0.246 | -43.4 | 0.466 | 95.8 | 0.323 | -141.5 | 0.866 | -220.7 | 0.757 |
| Senior high | -479.7 | 0.430 | -162.0 | 0.582 | -208.8 | 0.297 | -53.5 | 0.407 | 36.8 | 0.726 | 230.6 | 0.799 | -243.3 | 0.753 |
| Junior college and more | 172.1 | 0.796 | -145.3 | 0.651 | 22.9 | 0.917 | -58.5 | 0.407 | 126.1 | 0.273 | 1152.4 | 0.247 | 136.2 | 0.872 |
| **Job (baseline: Governments)** |  |  |  |  |  |  |  |  |  |  |  |  |  |  |
| Enterprises | 725.7 | 0.108 | -24.2 | 0.912 | -186.2 | 0.205 | -38.7 | 0.419 | -47.7 | 0.536 | 746.8 | 0.263 | 112.5 | 0.845 |
| Farmers | 1008.3 | 0.092 | 274.7 | 0.342 | -311.9 | 0.109 | -26.0 | 0.682 | 95.7 | 0.348 | 1271.0 | 0.153 | 292.3 | 0.701 |
| Small private business | 721.5 | 0.280 | 123.3 | 0.703 | -369.6 | 0.090 | -40.6 | 0.564 | -36.5 | 0.748 | 1548.2 | 0.114 | 783.5 | 0.350 |
| Others | 1320.4 | 0.043 | 192.9 | 0.542 | 64.0 | 0.764 | -72.2 | 0.306 | -18.3 | 0.871 | 2325.5 | 0.017 | 1482.9 | 0.078 |
| Retired | 2539.0 | <0.001 | 136.3 | 0.578 | 41.4 | 0.802 | -12.1 | 0.822 | -3.2 | 0.970 | 2592.6 | 0.001 | 1156.6 | 0.071 |
| No jobs | 1269.2 | 0.041 | 786.1 | 0.009 | -137.8 | 0.494 | 7.4 | 0.911 | -18.4 | 0.862 | 2268.3 | 0.015 | 1366.6 | 0.084 |
| **Areas (baseline: Rural)** |  |  |  |  |  |  |  |  |  |  |  |  |  |  |
| Urban areas | 594.6 | 0.142 | 372.1 | 0.058 | -16.1 | 0.904 | -12.2 | 0.777 | -188.2 | 0.007 | 320.3 | 0.592 | 41.7 | 0.935 |
| **Regions (baseline: Eastern)** |  |  |  |  |  |  |  |  |  |  |  |  |  |  |
| Central | -309.0 | 0.362 | -83.4 | 0.613 | 97.8 | 0.375 | -33.3 | 0.369 | 105.0 | 0.078 | 133.1 | 0.796 | 126.4 | 0.775 |
| Western | -763.4 | 0.016 | -107.1 | 0.483 | 87.0 | 0.398 | -46.8 | 0.158 | 23.7 | 0.657 | -848.5 | 0.067 | -570.2 | 0.150 |
| **Physical condition (baseline: Healthy)** |  |  |  |  |  |  |  |  |  |  |  |  |  |  |
| Just so-so | 494.8 | 0.093 | 24.5 | 0.863 | 149.8 | 0.117 | 5.916 | 0.849 | 82.4 | 0.100 | 788.5 | 0.069 | 817.9 | 0.028 |
| Slightly sick | 1635.7 | <0.001 | 509.2 | 0.011 | 268.8 | 0.047 | 53.641 | 0.223 | 106.0 | 0.134 | 2459.4 | <0.001 | 1672.5 | 0.002 |
| Sick | 1946.6 | <0.001 | 85.6 | 0.745 | 880.9 | <0.001 | 55.298 | 0.346 | 485.4 | <0.001 | 3590.7 | <0.001 | 3139.1 | <0.001 |
| Seriously sick | 1781.2 | 0.055 | -14.7 | 0.974 | 749.6 | 0.014 | -4.935 | 0.960 | 182.0 | 0.250 | 2780.0 | 0.042 | 2668.3 | 0.021 |
| **Household income (1K Yuan)** | 4.324 | <0.001 | -0.166 | 0.712 | 0.3 | 0.344 | 0.100 | 0.301 | -0.1 | 0.618 | 3.6 | 0.008 | -0.0 | 0.979 |
| **Personal income (1K Yuan)** | -3.8 | 0.169 | 0.5 | 0.733 | 0.3 | 0.756 | -0.1 | 0.666 | -0.2 | 0.712 | 2.8 | 0.496 | 6.1 | 0.079 |
| **Hospital (baseline: Grade Ⅰ hospital)** |  |  |  |  |  |  |  |  |  |  |  |  |  |  |
| Grade Ⅱ hospital | -28.4 | 0.937 | 29.3 | 0.866 | 98.4 | 0.398 | -22.6 | 0.553 | 79.9 | 0.194 | 416.4 | 0.434 | 506.4 | 0.267 |
| Grade Ⅲ hospital | 955.2 | 0.006 | 255.4 | 0.126 | 225.3 | 0.044 | 28.3 | 0.439 | 10.4 | 0.861 | 2079.1 | <0.001 | 1451.4 | 0.001 |
| Private hospital | 1073.6 | 0.045 | 120.6 | 0.642 | 34.6 | 0.844 | 35.1 | 0.543 | 173.9 | 0.062 | 881.2 | 0.274 | 468.3 | 0.493 |
| **Health insurance (baseline: not used)** |  |  |  |  |  |  |  |  |  |  |  |  |  |  |
| Yes | 756.9 | 0.005 | 167.9 | 0.197 | -67.4 | 0.440 | -9.926 | 0.727 | 92.5 | 0.044 | 1241.1 | 0.002 | -135.0 | 0.692 |
