# Supplementary material for: Health Insurance Utilization and Its Impact: Observations from the Middle-Aged and Elderly in China
Source: PLoS One. 2013 Dec 6;8(12):e80978. doi: 10.1371/journal.pone.0080978 (PMC3855696; doi:10.1371/journal.pone.0080978)
Supplement: Table S3 — Linear regression analysis of medical expenditure for self-treatment episodes. (DOCX) [file pone.0080978.s003.docx]

**Table S3: Linear regression analysis of medical expenditure for self-treatment episodes.**

|  | **Treatment** | | **Lost income** | | **Gross Total cost** | | **Out of pocket cost** | |
| --- | --- | --- | --- | --- | --- | --- | --- | --- |
|  | **B** | ***P*** | **B** | ***P*** | **B** | ***P*** | **B** | ***P*** |
| **Gender (baseline: Female)** |  |  |  |  |  |  |  |  |
| Male | -103.7 | 0.222 | 8.7 | 0.783 | -125.3 | 0.196 | -84.7 | 0.423 |
| **Age group (baseline: 45-50)** |  |  |  |  |  |  |  |  |
| 51-60 | 210.6 | 0.032 | -10.6 | 0.772 | 248.2 | 0.026 | 254.5 | 0.039 |
| 61-70 | 528.9 | <0.001 | -57.0 | 0.284 | 480.8 | 0.003 | 545.6 | 0.002 |
| >70 | 641.4 | <0.001 | -69.9 | 0.262 | 655.4 | 0.001 | 602.1 | 0.004 |
| **Marital status (baseline: Single/Divorced/Widowed)** |  |  |  |  |  |  |  |  |
| Married | -49.5 | 0.727 | 44.7 | 0.403 | 49.0 | 0.766 | 19.2 | 0.913 |
| **Education (baseline: No school)** |  |  |  |  |  |  |  |  |
| Primary | 39.1 | 0.820 | -22.8 | 0.717 | 67.9 | 0.726 | 83.1 | 0.675 |
| Junior high | 89.0 | 0.617 | 68.6 | 0.292 | 219.8 | 0.275 | 213.9 | 0.303 |
| Senior high | 83.3 | 0.665 | 16.8 | 0.812 | 153.1 | 0.480 | 123.6 | 0.585 |
| Junior college and more | -37.2 | 0.857 | 88.8 | 0.246 | 122.1 | 0.603 | 175.3 | 0.483 |
| **Job (baseline: Governments)** |  |  |  |  |  |  |  |  |
| Enterprises | -345.7 | 0.010 | 24.9 | 0.622 | -325.5 | 0.035 | -326.8 | 0.067 |
| Farmers | -464.2 | 0.011 | 166.1 | 0.014 | -338.2 | 0.101 | -287.8 | 0.198 |
| Small private business | -114.6 | 0.552 | 50.8 | 0.483 | -14.7 | 0.947 | 39.7 | 0.868 |
| Others | -406.2 | 0.037 | 136.8 | 0.062 | -249.4 | 0.265 | -147.0 | 0.550 |
| Retired | -533.1 | 0.001 | 54.2 | 0.368 | -517.2 | 0.005 | -432.9 | 0.037 |
| No jobs | -192.076 | 0.315 | 36.1 | 0.616 | -183.9 | 0.403 | -101.7 | 0.673 |
| **Areas (baseline: Rural areas)** |  |  |  |  |  |  |  |  |
| Urban areas | 348.831 | 0.006 | 16.4 | 0.726 | 380.7 | 0.008 | 426.3 | 0.005 |
| **Regions (baseline: Eastern)** |  |  |  |  |  |  |  |  |
| Central | 49.3 | 0.614 | 117.3 | 0.002 | 120.3 | 0.299 | 110.3 | 0.389 |
| Western | 31.5 | 0.751 | 58.2 | 0.104 | 63.0 | 0.566 | 7.1 | 0.953 |
| **Physical condition (baseline: Healthy)** |  |  |  |  |  |  |  |  |
| Just so-so | 259.6 | 0.004 | 39.4 | 0.233 | 318.1 | 0.002 | 380.2 | 0.001 |
| Slightly sick | 612.9 | <0.001 | 204.9 | <0.001 | 800.7 | <0.001 | 867.0 | <0.001 |
| Sick | 1185.6 | <0.001 | 276.0 | <0.001 | 1496.7 | <0.001 | 1451.8 | <0.001 |
| Seriously sick | 705.4 | 0.040 | -16.8 | 0.890 | 737.7 | 0.055 | 851.6 | 0.038 |
| **Household income (1K Yuan)** | 0.4 | 0.331 | -0.2 | 0.228 | 0.2 | 0.734 | 0.0 | 0.974 |
| **Personal income (1K Yuan)** | 1.6 | 0.054 | 0.0 | 0.919 | 1.8 | 0.056 | 1.6 | 0.157 |
| **Health insurance (baseline: not used)** |  |  |  |  |  |  |  |  |
| Yes | 49.4 | 0.597 | 28.5 | 0.410 | 51.1 | 0.632 | -141.9 | 0.287 |
